# Perception of racial discrimination in Brazilian school-aged children

**DOI:** 10.1186/s41155-025-00354-1

**Published:** 2025-06-05

**Authors:** Juliana Almeida Rocha Domingos, Luana Barretto Borges, Ana Carolina Messias, Débora de Hollanda Souza

**Affiliations:** 1 Graduate Program in Psychology, Universidade Federal de São Carlos, São Carlos, Brazil; 2 National Institute of Science and Technology On Behavior, Cognition and Teaching (INCT-ECCE), São Carlos, Brazil; 3 Department of Psychology, Universidade Federal de São Carlos, São Carlos, Brazil

**Keywords:** Racial and ethnic attitudes, Racism, Children

## Abstract

**Background:**

Fighting racial discrimination requires the ability to notice it when it occurs.

**Objective:**

The present study aimed to investigate the perception of racial discrimination in a sample of Brazilian school-aged children.

**Method:**

Fifty-three 6- to 12-year-old children were recruited from two public schools in a small town in the state of São Paulo, but there was no registration of the ethnic background of one child and, as a result, he had to be excluded. Therefore, the final sample consisted of 52 participants (10 black, 32 mixed-race, 10 white). A task designed to assess children’s perception of racial discrimination and used in previous studies was translated into Brazilian Portuguese and administered to participants. Children watched four videos of stories about an adult character who made a choice between a black child and a white child in different scenarios) (e.g., choosing a class leader, a student to represent the school in a science fair, a winner for the music contest and someone to complete the soccer team), with the choice always benefiting one over the other. Children were distributed into three conditions that varied in terms of whether racial discrimination was present or not. Two situational cues were manipulated: the skin color of the potential target of discrimination and information about the adult character’s past choices. At the end of each story, participants had to answer a question about the reasons for the choice made.

**Results:**

A significant effect of age was found on the PD task, but only for one condition (C1) when there was a pattern of apparent racial discrimination and when situational cues were provided, *U* = 21.0, *p* = .006. A D-prime analysis revealed that children were good at rejecting the existence of discrimination when it was not present; but they performed poorly when it was present, *d′* = − 0.44.

**Conclusion:**

These findings point to an important question regarding the when and how Brazilian parents and educators talk to children about ethnic-racial relations. This is an important future direction, and it can better inform intervention programs and public policy directed at preventing and fighting racism in our country.

## Introduction

Racial inequality has profoundly marked Brazilian society. More than a century has passed since the end of slavery in Brazil; however, the legacy of this painful period in our history, as well as the lack of public policy directed at promoting racial equality, still deeply affects black lives in this country (for a historical review, see Gomes, 2005; Munanga, 2004).

Public policy designed to fight racial inequality in Brazil is still timid. According to Gomes (2005), government institutions have repeatedly denied the existence of racism in this country based on the Myth of Racial Democracy, an ideology that has spread across the country since the 1930 s. As a result, many Brazilians believed (and still do) that ethnic-racial relations were harmonious and that racism did not exist in Brazil because we were a mixed ethnic group (Gomes, 2005). Thus, for a long time, not only was there no political agenda to prevent or punish racial discrimination, but racial stereotypes were perpetuated even further (e.g., explaining the economic and educational gap between whites and blacks in terms of merit and not due to the inequality of opportunities).

In fact, several statistics make abundantly evident the disastrous impact of racism in Brazil. Just to mention a few, according to the Brazilian Forum of Public Safety (2024), 78% of the victims of violent crimes in Brazil are black or mixed-race (“pardos”) and of the total number of deaths that were a result of police interventions, 82.7% were of black or mixed-race individuals. The average monthly wage of Brazilian white workers in 2021 was R$3.099.0 (approximately US$573.88, considering the average exchange rate in 2021) in contrast to R$1.764.0 (US$ 326.66) for black workers and R$1.814.0 (US$335.92) for “pardos” (Instituto Brasileiro de Geografia e Estatística [IBGE], 2022). Moreover, racial inequality starts early in life, as recent data suggests. Although 68% of white families have the financial resources to enroll their children in private daycare centers (“creches”; for children aged 0 to 3 years), only 32% of black families can afford those (AFRO-Centro Brasileiro de Análise e Planejamento [CEBRAP], 2023). Several studies have also described the experience of black school-aged children, in particular, the differential treatment they receive from white teachers, the pejorative nicknames they receive from their white peers and how perceived discrimination is found to be correlated with poor school performance (e.g., Matos & França, 2021; Moreira & Aguiar, 2015).

It is within this historic and cultural context of intense racial inequality that this study is justified, as it explores whether Brazilian school aged children recognize or perceive racial discrimination. If the existence of racial discrimination is denied in this country, or if adults feel the need to protect children from the painful realization of the negative impact of racial discrimination, will children be able to recognize it when it happens?

Recognizing such inequalities and understanding that systemic racism is at the base of such disparities is perhaps the most important step in the quest for an antiracist society (Gomes, 2005). Following this direction, psychological science can and should play a more active role in combating racism in Brazil, producing knowledge about how this complex phenomenon develops and how it impacts individuals and their relationships. It is exactly by means of this accumulated knowledge that we will be able to design and implement more effective interventions directed toward preventing and fighting racial discrimination, as well as helping those who fall victim to it. Despite some conceptual differences, there seems to be a consensus in the literature that education is vital to the eradication of racism (Cruz, 2016).

But how and when in development do children start detecting racism and racial discrimination? What are possible individual differences that explain variability in their capacity to recognize racial inequality in social contexts? The present work aimed to address these questions by investigating children’s ability to detect racial discrimination in different contexts. But to describe the study in more detail, a clear definition of racial discrimination needs to be provided.

Discrimination is conventionally defined as any biased behavior that directly brings harm or disadvantage to a member of a different group as well as any other action that unfairly favors a member of your own group (Dovidio et al., 2010). Such behaviors can be explicit (e.g., direct physical or verbal aggression) or implicit (e.g., giving more favorable treatment to an individual to the detriment of another). Racial discrimination is any discrimination based on race, but it should be distinguished from racial prejudice. According to Gomes (2005), discrimination entails behavior, whereas prejudice occurs at the cognitive level (e.g., beliefs and attitudes).

One promising and recent line of investigation focuses on parental ethnic-racial socialization (ERS) (Brown & Anderson, 2019; Brown et al., 2022; Nieri et al., 2024; Williams & Banerjee, 2021). This complex multidimensional construct captures how families conduct their children’s cultural socialization (i.e., how families socialize children about values, traditions, and practices associated with their ethnic–racial group); their preparation for bias (i.e., how families teach children about potential ethnicity/race based threats); promotion of mistrust (i.e., how families emphasize the need for wariness and distrust in interracial interactions); and how they teach strategies to cope with these experiences (Hughes et al., 2006).

Brown et al. (2022), for example, showed that ERS focus on racial discrimination is associated with latinx children’s ability to detect discrimination when it does happen. More specifically, children whose parents talked more about discrimination at home were more likely to report discrimination at school than children whose parents did not talk so frequently about the topic. Importantly, children did not present high levels of perceived discrimination in general, that is, they were only perceiving discrimination when it actually happened (better accuracy).

Therefore, ERS studies suggest that talking to young children about racial discrimination is an important step in the fight for racial equality. However, a systematic review of ERS literature (Nieri et al., 2024) indicates that white parents avoid discussions of ethnicity-race with their children, especially at an earlier age. They often adopt colorblind racial socialization strategies that discourage discussion about race. Moreover, they are more likely than parents of color to use strategies of advantage that reinforce their privilege and structural inequities. There is also evidence of an age effect in other studies. For example, Williams and Banerjee (2021) found that parents with older children were more likely than parents with younger children to discuss ethnic/racial discrimination.

A systematic review of studies on racial prejudice (Sacco et al., 2016) has shown that the topic has gained much interest from Brazilian researchers between 2009 and 2014, especially from Social and Developmental psychologists. The focus of these studies varied: effects of racism on mental health; the history of racism in Brazil; how Psychology can contribute to the eradication of racism; and Brazilians’ attitudes toward affirmative action policies. Although Sacco et al. (2016) point to the important progress made in psychological research on the topic, they argue that more studies investigating the development of racism in young children are needed.

Matos and França (2023), in turn, conducted an integrative review of studies investigating the different forms of racism found in schools. Sixteen articles met all the inclusion criteria and they were categorized according to the following seven themes: (a) racism in early education; (b) in documents and books frequently used for teaching; (c) in religion teachings; (d) in higher education; (e) in secondary education; (f) in the educational reform of the state of Bahia; (g) racism as a predictor of school failure in early elementary school. According to the authors, in all these different contexts, the forms of racism range from lack of attention devoted to black students, to negative labels (e.g., “out of control”) to the silence and omission of racist attitudes or discriminatory behavior.

More recently, Sacco et al. (2019) investigated both implicit and explicit racial attitudes in a large sample of white, mixed-race (“pardo”), and black children and adolescents (6- to 14-year-olds) from two Brazilian states with distinct sociodemographics: Bahia and Rio Grande do Sul. Whereas in Bahia, the majority of the population is either black or “pardo,” in Rio Grande do Sul, the pattern is just the opposite, with 84% of the population being white. Thus, this comparison provided a nice test of the possible effects of racial diversity in racial attitudes. Contrary to their initial prediction, participants from Bahia showed stronger pro-white bias, regardless of their own racial group. In contrast, participants from RS were more likely to identify themselves with their own group. Overall, their findings suggest that diversity alone may not be sufficient to explain variability in racial prejudice among children and adolescents; but other variables such as the social and economic status of each racial group need to be taken into consideration. But even before children manifest racial bias or explicit prejudice, they should be able to recognize when racism or racial discrimination occurs.

According to Brown and Bigler (2005), children’s ability to perceive discrimination develops as they acquire important cultural and social-cognitive skills. For example, to perceive discrimination, children have to understand that individuals can be grouped into categories based on socially defined characteristics (e.g., gender or race); that there are stereotypes associated with these social categories; and that the use of these stereotypes leads to important implications. Such understandings are part of what they call cultural cognition.

Additionally, theory-of-mind development needs to be in place. A prerequisite for detecting racial discrimination is the understanding that other people have their own thoughts, intentions, and beliefs, which may be different from their own, and that these mental states underlie their behavior. As social cognition develops, children become capable of realizing that one’s thoughts may be incongruent with their actions. Later, in adolescence, they begin to understand that society reflects prejudiced thoughts of individual members (Brown & Bigler, 2005). Other cognitive skills are also required, such as multiple classification skills, moral reasoning, the ability to make justice judgments based on the principle of equity rather than equality, as well as the understanding that social systems produce inequities between social groups.

Children also rely on a set of situational cues, which may increase or decrease the chance of someone perceiving the occurrence of discrimination. One cue, for example, is social comparison, when available (e.g., treatment given to black individuals x that given to white individuals). Another important cue is data on one’s past attitudes (e.g., a history of discriminatory behavior). A third situational cue is related to whom discrimination is targeted at. More specifically, it is easier to perceive discrimination when somebody else is the target, mainly because being a victim of discrimination may trigger negative feelings (Brown & Bigler, 2005).

Some cognitive skills are prerequisite for children to perceive such situational cues, however. For example, the presence of members of groups with different statuses in a situation of differential treatment to a member of a stigmatized group will only be used as a situational cue by children who are already capable of making social comparisons. Likewise, knowing a teacher’s history of discrimination will not be understood as a situational cue in the context of classroom discrimination, if the child has not acquired cultural cognition skills, or even the basic understanding of moral reasoning (i.e., that people can act unfairly) (Brown & Bigler, 2005). Finally, Brown and Bigler (2005) describe some individual differences that may explain why some children are better at perceiving than others: the amount of accumulated knowledge about discrimination, having ERS offered by parents and a strengthened ethnic identity, etc.

Although Brown and Bigler’s model has been consistent with other studies conducted with US children (e.g., Brown, 2006), the specific way in which racism manifests itself in Brazil does not allow for a mere import of such a model. Considering the potential benefits of similar studies with children from other cultural backgrounds, as well as the robust evidence on the negative impact of racial discrimination, the present work aimed to investigate when and how the perception of discrimination develops in a sample of Brazilian school-aged children. More specifically, the present study aimed to analyze whether situational cues (e.g., information about the racial attitudes of those who engage in discriminatory behavior and the color of the person who was the target of racial discrimination) facilitate children’s perception of such discrimination. Finally, we tested for a possible effect of their perceived ethnic-racial background on the perception of racial discrimination.

### Method

The design of the current study was cross-sectional as it tested for possible differences between two age groups (6–8- and 9–11-year-olds), and quasi-experimental as the main goal was to test for a possible effect of condition on children’s performance in the perception of discrimination task (PD task).

#### Participants

Fifty-three school-aged children (26 boys and 27 girls) were recruited from a public school located in the state of São Paulo for the present study. However, there was no registration of the ethnic background of one child and, as a result, he had to be excluded. Therefore, the final sample consisted of 52 participants. Nine participants self-identified themselves as black (17.3%), 32 self-identified themselves as mixed race (61.5%), 9 self-identified themselves as white (17.3%); one child said she did not know what her ethnic-racial background was, but the experimenter registered her color as being white; and another child said he was white, but he was black. Children were divided into two age groups: 31 participants attending second and third grades (*M*_age_ = 7 years and 7 months; *SD* = 8.8 months) and 21 participants attending fourth and fifth grades (*M*_age_ = 10 years and 3 months; *SD* = 10.7 months).

#### Data collection

This research was approved by the Ethics Committee on Human Research of Universidade Federal de São Carlos, in accordance with Resolution n^o^ 510/2016, which is based on the Universal Declaration of Human Rights and the American Declaration of The Rights and Duties of Man. Participants were recruited from a public elementary school. First, after being introduced to children by the teacher, one of the experimenters talked about the research project and answered any question children might have. The research was presented to each child without mentioning the research question and with no use of ethnic-racial terms to avoid prompting children’s responses during the session. Children were informed that the purpose of the study was to “investigate what and how children think.” The Informed Consent Form was sent to their parents. The main goal of the research project and a brief description of the tasks to be held during the experimental session were described in the Informed Consent Form. Only children whose parents signed the consent form were invited to participate. The experimenter always read the Informed Assent Form with the child, and if they agreed to participate, the experimenter would ask them to sign the Informed Assent Form. Data collection was conducted individually in a quiet room in the school by one of two female experimenters (one black and one white). Each experimental session with a child lasted approximately 20 min.

#### Instruments

Racial categorization task (RC task). The experimenter showed 12 pictures of children’s faces in random order (4 black, 4 mixed-race, and 4 white). Children were asked to identify the skin color of each face, presented on a computer screen. For each correct answer, they received a score of 1 point and thus, the final score ranged from 0 to 12 points. Finally, children were asked about the skin color of the experimenter (“What do you think my skin color is?”). They received 1 additional point if they answered correctly and 0 point, if the answer was incorrect (e.g., saying the black experimenter was “white”).

Racial self-identification task. Initially, participants were asked an open question: “What color are you?” In case the child did not provide an answer or if they said they did not know, the experimenter presented the following options: white, mixed-race, or black. If the participant gave an unusual answer (e.g., “My color is caramel”), they were asked in which category that fell (e.g., “Caramel color is white, mixed-race or black?”). The main goal of this task was to analyze children’s self-perception of their ethnic-racial background. We also intended to test for a possible effect of their perceived ethnic-racial background on their ability to detect racial discrimination.

Perception of racial discrimination task (translated and adapted from Brown, 2006). To assess the perception of racial discrimination, participants were presented with four short stories, in a video format. All of them involved a situation during which an adult had to make a choice between two individuals (i.e., choosing a class leader, a student to represent the school at a science fair, a winner for the music contest and someone to complete the soccer team), but each choice always favored one to the detriment of the other. During storytelling, participants were informed that the characters were equally talented or performed equally well. Thus, the only difference between them was their skin color: one character was white, and the other one was black, but such difference was never brought up in conversation.

Two situational cues were manipulated. The first one was the color of the potential target of discrimination, that is, either the potential target of discrimination was the black child (e.g., the teacher, who was a white adult woman, always chose a white child, in detriment of a black child) or the white child (e.g., a black teacher always chose a black child, in detriment of a white child). As a result, two stories favored a white child and two favored a black child.

The second situational cue was information about the previous history of the adult character’s choices. In consonance with Brown’s model (2006), we predicted that the adult’s prior choices could be indicative of their racial attitude. More specifically, if they always chose children of the same ethnic-racial group as theirs, it would be a strong indication of a more negative attitude toward members of other ethnic-racial groups.

**Fig. 1:**
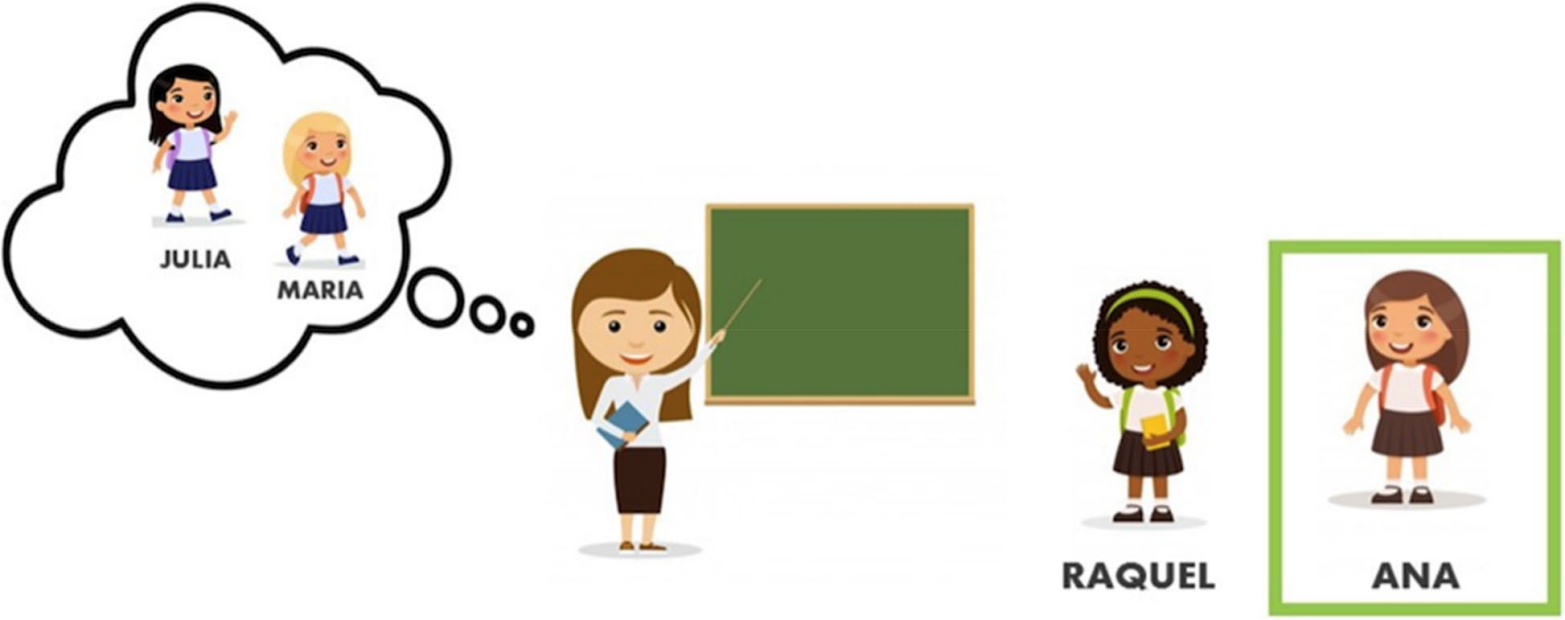
Scene from the first story (C1)

Participants were distributed into three conditions. In Condition 1 (C1), there was a pattern of apparent racial discrimination, with the adult character always choosing a child of the same skin color as his/her (see Fig. 1). For half of the stories, the discrimination target was a black child and for the other half, the discrimination target was a white child. Furthermore, information about the adult character’s past choices was provided (e.g., “Last year, X was chosen; and the year before, Y was chosen!”). In Condition 2 (C2), there was no discrimination pattern in the stories (e.g., a white teacher chose a white child in the present but had chosen a black child in the past). In Condition 3 (C3), no information about the adult character’s past choices was provided. There were two stories with a clear discrimination pattern (i.e., an adult chose a child of the same color as his/hers) and the other two stories revealed no discrimination pattern (i.e., an adult chose a child of a different color than his/hers).

At the end of each video, participants were asked to answer an open question about why the adult had chosen that child. For the stories that involved racial discrimination (C1 and C3), children received 1 point when they perceived discrimination and 0 point when they had a different explanation (e.g., “Because Ana was nicer!”). For the stories that did not involve racial discrimination (C2 and C3), children received 1 point when given alternative explanations, and 0 points when they alleged racial discrimination was involved. The total score for this task varied from 0 to 4.

#### Procedures

The Perception of RD task was administered first to avoid a possible effect of exposure to information about racial categories. The RC task was administered next, followed by the Self-Identification measure.

The experimenter also held a debriefing session with each child, explaining the purpose of the study. The main goal was to check whether the procedure had any emotional impact on them. More specifically, after completing all tasks, the experimenter asked the child: “How are you feeling right now?” Only one child (P25, 9 years and 3 months, self-identified as mixed-race (“parda”) expressed feeling sad when hearing the stories involving racial discrimination. In this instance, the experimenter talked to her about her feelings, validating her emotions until the child felt better. At the end of each session, all children heard the experimenter saying that every person is unique and that, regardless of skin color or any other possible differences, everybody deserves to be treated with respect and kindness and is capable of being good and talented. They also watched a video clip of animated characters singing a song about the importance of diversity, and how everybody deserves love and respect, and that differences should always be welcomed. It is also important to note that previous studies using this same task (Brown, 2006) and very similar procedures to assess children’s intergroup attitudes (Brown, 2011; Brown et al., 2017) did not find any negative effects of children’s participation in the studies.

## Results

First, we present descriptive data on children’s performance in the PD and RC tasks (Table 1). Shapiro–Wilk tests revealed that score distributions were not normal (*ps* = n.s.); as a result, we opted for non-parametric tests. A trend toward a significant gender effect was found for the RC task (with age collapsed), *U* = 240.0, *p* = 0.07, but no significant difference between boys and girls was found on the PD task, *p* = n.s. Therefore, gender was not included in subsequent analyses.

**Table 1:** Participants’ performance on the PD task and RC tasks: mean (SD)

		**PD**	**RC**
**C1**	6- to 8-yr olds	0.0 (0.0)	10.2 (0.9)
	9- to 11-yr olds	1.3 (1.5)	11.3 (0.8)
**C2**	6- to 8-yr olds	3.6 (1.3)	9.7 (2.8)
	9- to 11-yr olds	4.0 (0.0)	10.4 (1.8)
**C3**	6- to 8-yr olds	2.0 (0.0)	8.3 (2.3)
	9- to 11-yr olds	2.4 (0.5)	9.7 (1.9)

As can be seen in Table 1, children from both age groups performed well on the RC task, with a trend toward a significant difference found between them, *U* = 230.5, *p* = 0.07. To explore if children were able to correctly identify the ethnic-racial background of the experimenters, we conducted a Mann–Whitney test which revealed no significant difference in their classification skills (answer to the question “What do you think my skin color is?”) when data collection was conducted by the white experimenter versus when it was conducted by the black experimenter. No correlation was found between children’s scores on the RC task and those on the PD task, *r*_*s*_ = − 0.14, *p* > 0.05.

A significant effect of age was found on the PD task, but only for C1, with older children performing better (*M* = 1.3; *SD* = 1.5) than the younger group (*M* = 0.0; *SD* = 0.0), *U* = 21.0, *p* = 0.002. No age difference was found in C2 and C3, *U* = 28.0, *p* > 0.05 and *U* = 16.0, *p* > 0.05, respectively. A Wilcoxon signed rank test revealed, however, that children’s overall performance on the PD task (with children from all three conditions) was not different from chance both for the younger group (*p* = 0.14) and the older group (*p* = 0.1).

For C3 (two trials with a clear discrimination pattern and two with no discrimination involved), we ran a D-prime analysis to test children’s sensitivity to the difference between situations when discrimination is present and those when it is absent (see Macmillan, 2002). First, mean hit rates (*M* = 0.20) were calculated, that is, the proportion of times participants detected racial discrimination when it was present; and next, false alarms (*M* = 0.10), or the proportion of trials during which they detected discrimination when it was not present, were calculated. The d′ value was − 0.44, suggesting children’s low sensitivity to the difference between the presence and absence of discrimination. More specifically, hit and false alarm rates suggest that children do better when they are presented with stories that do not involve racial discrimination as they correctly reject its existence. In contrast, when it is present, they fail to detect it.

Next, we grouped participants’ answers to the open questions into eight categories according to content, namely: (1) in-group bias (e.g., “They have the same skin color,” “The teacher has a different skin color than the student”); (2) prejudice-general (e.g., “It might be because she sang better, or because of prejudice”); (3) prejudice-skin color (e.g., “Because of the skin color, or hair differences,” “Because she is black”); (4) inequality (e.g., “In the past, she chose a child of a different skin color”); (5) avoiding racism (e.g., “The teacher suffered racism and chose the white student because she wanted to prevent the black one from suffering racism if she didn’t do well”); (6) competence/attitudes (e.g., “The best,” “The smartest,” “The best grade,” “Sang better,” “Least behaved”); (7) random or alternate choice (e.g., “Alphabetical order,” “Because the teacher wanted that,” “Next time, she’ll pick the other kid”); (8) No clear explanation (e.g., “The name of the child who was picked by the teacher is a good fit for soccer”). As shown in Table 2, the most frequent answer to justify the character’s choice concerned the child’s competence or attitudes.

**Table 2:** Frequency and percentage of the answers by category

Category	Frequency	%
In-group bias	6	2.8
Prejudice-general	6	2.8
Prejudice-skin	5	2.4
Inequality	2	0.9
Avoiding racism	3	1.4
Competence/attitudes	172	81.1
Random or alternate choice	14	6.6
No clear explanation	4	1.9
Total	212	100

A Mann–Whitney test did not reveal a significant difference in children’s performance when stories showed a previous history of racial discrimination (C1) versus when there was no such information (C3), *U* = 142.5; *p* > 0.05. Additionally, we did not find a significant difference between scores in stories without racial discrimination but showing the character’s past choices (C2) and stories with no racial discrimination and no information about past choices (C3, stories 2 e 3), *U* = 105.0, *p* > 0.05. Finally, a Wilcoxon test did not show a significant difference between C1 mean scores for the subset of trials during which the target of racial discrimination was a white child (*z* = − 1.0) and for those during which the target was a black child, (*z* = 0.0), *p* = *n.s*.

Finally, we wanted to test for a possible effect of perceived ethnic-racial background (i.e., self-identification) on their ability to detect racial discrimination. Mixed-race participants were excluded from this final analysis for two main reasons. First, the number of children who identified themselves as mixed-race was more than three times larger (*n* = 32) than those who identified themselves as white (*n* = 9) or black (*n* = 9). Additionally, two children were excluded from these tests because one could not name their skin color, and the other reported having a skin tone different from the color perceived by the researcher. A Mann–Whitney test did not reveal a significant difference in performance on either the PD task, *U* = 32.5, *p* > 0.05, or the RC task, *U* = 28.5, *p* > 0.05.

## Discussion

The main goal of the present study was to investigate the perception of racial discrimination in a sample of Brazilian school-aged children. More specifically, we aimed to understand possible effects of cognitive, situational, and individual aspects on children’s ability to detect racial discrimination. The present work also investigated a possible age effect, as shown in previous studies (Bigler et al., 2008; Brown & Bigler, 2005). It is important to note, however, that our focus was not on racial discrimination/prejudice per se, or even children’s racial bias or attitudes, but on children’s perception of racial discrimination. To do that, we largely drew from Brown and Bigler’s work (2005) on children’s perception of gender discrimination. Their work, like ours, is in line with Bigler and Liben’s developmental model (2006) of the formation of stereotyping and prejudice among children.

Our results suggest that older children (9- to 12-year-olds) are better than younger children (6- to 8-year-olds) at detecting racial discrimination when it is present and when situational cues regarding past behavior are provided (C1). A similar age effect was found in Bigler et al. (2008). Children from different racial backgrounds and with ages ranging between 5 and 10 were asked to explain why there had never been a woman or an African American/Latino president in the USA. Older children presented more explanations based on racial discrimination (e.g., “White people didn’t like Black people,”; “White people don’t want a Black person to be president,” and “George Bush and other presidents hate Black people”), when compared to younger children.

Another study (McKown, 2004) explored children’s thinking about racism in an ethnically diverse sample of 6- to 10-year-old children in the USA. Older children provided more elaborate and differentiated definitions of racism, which is consistent with our findings. Moreover, African American children in this study were more likely than other racial groups (White, Asian, and Latino children) to talk about discrimination and ethnic conflict and were more likely to define racism as a form of putting down outgroup members, which, according to McKown, may “reflect a precocious awareness of power as a salient dimension of racism” (p. 613).

Sociocognitive development, in fact, explains part of the variability in children’s perception of discrimination. For example, Brown (2006) found that children with a more sophisticated theory of mind are more likely to attribute racial/ethnic bias as the underlying cause of a prototypical discrimination scenario, whereas younger children, with a yet limited understanding of others’ mental states, are more likely to claim physical and observable attributes, such as skin color, to be the cause of discrimination.

Nonetheless, previous studies show that most of the social cognitive abilities required for the detection of discrimination are developed by age 7, and children this age are already capable of perceiving more explicit forms of discrimination (Brown, 2017). According to Brown and Bigler’s model (2005), on the other hand, older children (8–10-year-olds) present a more refined comprehension of how racial discrimination works and how it impacts victims. In contrast to younger children, their moral reasoning includes the realization that: (a) discrimination violates ethical and moral values (fairness principle); and (b) that people may act unfairly. Finally, older children start to make fairness judgments based on the principle of equity, rather than on the principle of equality. One more important achievement that emerges at ages 8 or 9 is the ability to make comparisons between, within and across social groups. In other words, older participants in our study may have compared the performances of the two child characters in the stories and noticed that the teachers’ choice in the discrimination scenarios could only be explained by racial bias. One interesting future direction for the present study is to investigate possible associations between theory of mind and the perception of racial discrimination in Brazilian school-aged children.

Brown (2006) also found a low percentage of discrimination attribution responses among participants (9.6%), who ranged in age from 5 to 11 years. Additionally, 57.1% of the children in her study provided answers related to a possible difference in the quality of work presented (e.g., “He hit the ball farther”; “He was a better singer”), disregarding the possibility of differential treatment due to discrimination. Likewise, when we look at our participants’ justifications for the teacher’s choices, only 10.4% of their reasons are related to racial/ethnic bias. Nonetheless, according to Brown and Bigler (2005), 10-year-olds have all the necessary requirements to correctly infer whether racial/ethnic discrimination is present or not, just like any typically developing adult. Future studies should investigate further the ability to perceive discrimination in older children (10-year-olds and over) to better understand the discrepancy in such findings.

Another important goal of the present study was to test for possible effects of cultural cognition, or the ability to understand that individuals can be grouped into categories based on socially defined characteristics (e.g., gender or race). Past studies have shown that around age 6, children are already capable of categorizing people, including themselves, according to their race (Brown & Bigler, 2005). In line with such predictions, our participants performed very well in the RC task. Surprisingly, we did not find a correlation between children’s performance in the RC task and in the PD task. This pattern of results suggests that being able to categorize people into racial groups may be necessary but not sufficient to the perception of racial discrimination.

In fact, cultural cognition also involves recognizing the existence of stereotypes associated with racial/ethnic categories and their impact. According to Brown and Bigler (2005), understanding that there are stereotypes associated with racial categories increases the probability of children identifying discrimination. They bring up evidence suggesting that, at age 6, children already understand the social implications of stereotypes and this understanding becomes better with age. Therefore, another promising direction is to explore children’s understanding of stereotypes and its contributing role to their ability to detect racial/ethnic discrimination.

Contrary to what was expected, our results did not reveal an effect of situational cues, such as knowledge about the teacher’s history of discriminatory behaviors and the color of the target of discrimination. Brown (2006), on the other hand, found that the color of the target of discrimination had an effect on participants’ perception of discrimination, with performance being better when the target was a latinx child and the information about past choices indicated a consistent choice for a white child. One important piece of information that might help explain these different findings is the amount of talk parents have with their children about ethnic/racial discrimination. It could be that children from our sample were not very good at detecting discriminatory behavior because when it happens around them, nobody talks about it. Brown et al.’s findings (2022) are consistent with this hypothesis, as they suggest that talking about discrimination is correlated with children’s ability to detect discrimination when it actually occurs. Future studies should explore parental attitudes toward conversations with their children about racial discrimination.

In fact, Hughes et al. (2007) demonstrated the importance of teaching racial issues to children. The study was conducted with white children (study 1) and black children (study 2) with ages between 6 and 11 years old, who were presented with lessons about racism during 6 days. The results indicated that, for both white and black children, learning about racism had positive effects. White children acquired more positive racial attitudes toward black people, whereas black children had an improvement in racial attitudes toward black people, showing high levels of satisfaction with the lesson. These findings demonstrate the importance of interventions aimed at teaching about racial discrimination in schools and the implementation of policies to combat racism (see also Wilton et al., 2023).

Another possible explanation for the fact that our participants did not seem to attend to the situational cues provided is related to their own prior experiences with discrimination. Children were recruited in public schools which are known to have an ethnic-racial distribution that is more diverse and more representative of the one we find in the Brazilian population, in general. In private schools, however, the majority of students are white and from middle- to upper-class families. One could argue that situational cues were not perceived by most of our participants simply because they have had less experience witnessing racial discrimination, although that remains a speculation. Recent evidence, however, suggests that racial diversity alone is not sufficient to explain variability in children’s racial attitudes (Sacco et al., 2019). An interesting future direction would be to include children from both public and private schools and test for possible differences between these two groups. In addition, it would be informative to have both a measure of children’s ability to perceive discrimination in different scenarios and a measure of their racial bias.

The results from the present study also showed that black children were not better than white children at identifying racial discrimination, which is surprising, according to the model proposed by Brown and Bigler (2005). However, other studies did not reveal effects of racial belonging on perception of discrimination (Bigler et al., 2008; Brown, 2006; Elenbaas & Killen, 2017). It is possible that discrimination experienced by children themselves is a better predictor of their ability to perceive racial discrimination than racial belonging (Elenbaas & Killen, 2017).

The present study has some limitations. First, we acknowledge the sample size is rather small and unfortunately, we were unable to get a more diverse sample, with similar numbers of participants in each racial category. Although the number of recruited white children is indeed small, the proportion of mixed-raced participants (61.5%) from our study is not that distant from that found in the population of children attending public schools (53.7%). It is also important to note that the Instituto Nacional de Ensino e Pesquisas Educacionais Anísio Teixeira (INEP) school census (2023), which is collected annually, reveals there is still a significant percentage of unreported data on race (25.5%), which is a challenge for those who need accurate sociodemographic data of school-aged children. INEP is the Brazilian federal agency (affiliated with the Ministry of Education) responsible for compiling and analyzing data from education institutions and operates in three main areas: educational assessments and exams; statistical research and educational indicators; and data management. Nonetheless, future studies should aim to get a more representative and diverse sample.

Another limitation of the present study is that, although we did not find any signs of negative effects on participants, we should have planned a follow-up session with them and the school where recruitment was conducted. This follow-up session could have provided important evidence of whether the study contributed positively to children’s understanding of racial discrimination. At the same time, there is still significant reluctance from parents, educators and researchers to even start a conversation about racial bias with children. But, as Brown and Anderson (2019) put well: “there’s one big problem with this trepidation: children see identity. They see it on their skin, their hair, their clothes, their voices. They talk about it, experience it, and interact with others based on race and gender” (p.1). Future studies should contemplate the possibility of not only assessing participants’ perspectives on ethnic-racial relations but also establishing an opportunity for the family and the school agents to talk about racial discrimination.

A third limitation of the present study is that the task used to measure perception of racial discrimination was originally designed for a study conducted in the USA. Therefore, it is still possible that the task is simply not sensitive enough to measure the perception of racial discrimination in Brazil. In future studies, it would be helpful to add additional measures and test for possible correlations.

## Conclusion

Our work presents unprecedented data on the perception of discrimination in a sample of Brazilian school-aged children. More studies, however, are needed if we want to better understand the development of the perception of racial discrimination in Brazilian children, especially because we need data to support intervention programs and public policy aimed at reducing racial discrimination in this country. It is by understanding how children perceive and deal with discrimination today that we will be able to find effective ways to prevent it from happening tomorrow.

In particular, school communities exert a major influence on children’s beliefs about race and difference (Smith et al., 2003). Schools are important landmarks for population-based programs directed at fighting racism as they help students learn how to identify, resist and cope with racial discrimination. Additionally, some studies suggest that the most effective school interventions are those grounded in developmental theory and evidence, targeting not only individual-level attitudes and beliefs but also structural, systemic, and institutional changes (Beelmann & Heinemann, 2014; Bigler & Wright, 2014; Walton et al., 2013).

For example, a recent study evaluating the effects of an anti-racist program in primary schools showed that providing resources and structured lessons to teachers gives them a platform to raise the topic in class with more credibility, as some of them report having doubts about what to say and about how to introduce the topic to students during classes (Priest et al., 2021). Families also play a major role in children’s ERS, more specifically, in their developing ability to recognize, confront, and cope with racial discrimination. How parents discuss and perceive discrimination significantly predicts children’s early awareness of such issues (Brown & Bigler, 2005).

To foster children’s understanding of discrimination at an early age, parental ERS should incorporate preparation for bias (Brown et al., 2022) that will help them to recognize and cope with racial discrimination (Hughes et al., 2006). However, adults may hesitate to engage in preparation for bias discussions, possibly to shield children from the negative aspects of ethnicity (Huguley et al., 2019). Many conversations about discrimination tend to be reactive, occurring only after a bias incident has taken place (Brown et al., 2022). On the other hand, studies have shown that engaging in ERS preemptively offers stronger psychological protection than reactive interventions, such as discussions after children have experienced or witnessed racial discrimination (Derlan & Umaña-Taylor, 2015; Thomas et al., 2009). In sum, early talk about ethnic-racial relations may be more beneficial and protective for children (Anderson & Stevenson, 2019).

Lastly, most participants in the present study failed to detect racial discrimination even when situational cues were present. Although many, if not all of them, have already witnessed or suffered from different forms of racial discrimination, it is surprising that they do not recognize it in the stories we provided. One possible explanation relates to the challenges we face to effectively socialize children about race and racism (Wilton et al., 2023). For example, we need teachers who are better prepared to do so. Educators need adequate training, during their formative years in college, to do so as professionals, but much progress is still needed on that front (Afro-CEBRAP, 2003; Silva & Cruz, 2022). At home, parents also need to be prepared to engage in informative conversations with their children about race and racial discrimination (Brown, 2019; Wilton et al., 2023). To the extent of our knowledge, there is still little work investigating how Brazilian parents from different racial groups and from different socioeconomic backgrounds talk to their children about ethnicity/race, and if so, what are the topics being addressed, as well as when and how they are introduced. This is an important future direction of the present work, as it may shed light on the contextual factors that contribute to young children’s ability not only to recognize, but also to fight racial discrimination in their environment.

## Data Availability

The datasets used and/or analyzed during the current study are available from the corresponding author on reasonable request.

## Abbreviations

C1: Condition 1
C2: Condition 2
C3: Condition 3
RC task: Racial categorization task
PD task: Perception of discrimination task
ERS: Ethnic-racial socialization
IBGE: Instituto Brasileiro de Geografia e Estatística
INEP: Instituto Nacional de Ensino e Pesquisas Educacionais Anísio Teixeira
